# Genetic Insights on Hypertriglyceridaemia‐Induced Acute Pancreatitis in Pregnancy: A Case Series and Literature Review

**DOI:** 10.1155/crie/5330997

**Published:** 2026-02-09

**Authors:** Christopher Son Nguyen, Nicholas Adams, Emily Gianatti, I-Lynn Lee, Dev Kevat

**Affiliations:** ^1^ Department of Endocrinology and Diabetes, Western Health, St. Albans, Victoria, Australia, westernhealth.org.au; ^2^ Department of Endocrinology, Fiona Stanley Hospital, Murdoch, Western Australia, Australia, fsh.health.wa.gov.au; ^3^ Department of Medicine, University of Melbourne, St. Albans, Victoria, Australia, unimelb.edu.au

**Keywords:** genomics, hypertriglyceridaemia, pancreatitis, pathogenic variants, pregnancy

## Abstract

**Context:**

Hypertriglyceridaemia‐induced pancreatitis in pregnancy (HTG‐IPP) is a rare but serious condition. There is a paucity of evidence‐based guidelines and recommendations for screening and management of HTG‐IPP. Individual genomics can predispose certain populations to a higher risk of developing HTG‐IPP.

**Objective:**

To report on a case series of the management of four individual pregnancies complicated by HTG‐IPP, subsequently found to be associated with pathogenic genetic variants involved in triglyceride (TG) metabolism.

**Methods:**

The medical records of four individual pregnancies from two metropolitan hospitals in Australia were reviewed regarding the management of their HTG‐IPP and genetic testing for hypertriglyceridaemia (HTG). A literature review of previous cases of HTG‐IPP with an identified pathogenic variant was performed.

**Results:**

The identified genetic variants resulting in a diagnosis of HTG and HTG‐IPP were within glycosylphosphatidylinositol anchored high density lipoprotein binding protein 1 (GPIHBP1), lipoprotein lipase (LPL) and apolipoprotein A5 (APOA‐5). All patients had co‐morbid gestational diabetes mellitus (GDM) and were of South Asian or Asian ethnicity. All four patients were effectively managed with fasting, intravenous insulin, omega‐3 fatty acids (O3FAs) and very low‐fat diet (VLFD) with supplementation with medium‐chain TG (MCT) oil.

**Conclusion:**

Further genomics research is needed to increase our understanding for its use in predicting risk of severe gestational HTG. With additional case identification, particular variants of pathogenic interest can be identified and screened for antenatally in patients with a moderate fasting HTG of more than 200 mg/dL (11.1 mmol/L) in the absence of other causative factors. Pre‐conception optimisation of TGs and regular monitoring in pregnancy can reduce the incidence and disease burden associated with HTG‐IPP and HTG.

## 1. Introduction

Gestational hypertriglyceridaemia (HTG) is defined as plasma triglyceride (TG) >95th percentile for age during pregnancy whilst severe HTG is defined as TG >11.4 mmol/L [1]. Gestational HTG is associated with an increased risk of acute pancreatitis, pre‐eclampsia and hyperlipoproteinemia post‐partum [2–4]. HTG‐induced pancreatitis in pregnancy (HTG‐IPP) is historically associated with high maternal and fetal mortality (21%–37% and 11%–37%, respectively), but with improvements in screening, earlier diagnosis and intervention mortality rates have decreased (1% and 0%–18%, respectively) [5, 6]. The incidence of pancreatitis in pregnancy is 3–10 in 10,000 pregnancies [3, 6, 7]. Biliary obstruction followed by alcohol‐induced pancreatitis were traditionally considered the most common causes; however, recent evidence suggests HTG may be just as common and is the most common cause in those of Chinese ethnicity [6–8].

There is limited data pertaining to genomics of HTG‐IPP; however, studies to date indicate that there is an underlying genetic mutation in most cases [1, 9–17]. HTG‐causing rare variants in lipoprotein lipase (LPL), APOC2, APOE, GCKR, GPIHBP1, and LCAT genes have been identified. Other mutations that have been linked with chylomicronemia include those in LMF1, apo C‐II, apo A‐V, and GP1HP1 [18]. Familial chylomicronaemia syndrome (FCS) itself, with the presence of bi‐allelic mutations leading to LPL deficiency and the absence of activating proteins, is rare in clinical practice with an estimated incidence of 1 in 1,000,000 [19].

The concentrations of all lipoproteins increase in normal pregnancy. Very low density lipoprotein and TG increase 2.5 times, low density lipoprotein increases 1.6 times, while high density lipoprotein increases to 1.5 times above normal before declining to 1.2 times at full term [1]. These changes are mediated primarily by progesterone, oestrogen and human placental lactogen (HPL) as well as increased substrate from exogenous intake related to increased appetite. The changes impact TG levels throughout pregnancy but are more pronounced as the pregnancy progresses. The changes are usually of no clinical relevance; however, in the setting of a genetic susceptibility towards HTG, this can become pathological. Non‐genetic causes of HTG in pregnancy are less common but include diabetes, excessive alcohol intake, renal disease, beta blockers, thiazide diuretics and corticosteroids.

Clinical guidelines for management of HTG‐IPP are lacking and there is an overall paucity of high‐level evidence. Current evidence is drawn from individual case reports and limited case series.

This study is a retrospective case series of HTG‐IPP occurring in four separate pregnancies amongst four patients over a 7‐year period between 2015 and 2021 who presented to two specialist maternity hospitals in metropolitan Australia. This case series focuses on the genetic mutations identified to guide ongoing management and counselling for future pregnancies.

## 2. Methods

Recurrent episodes of acute pancreatitis within the same pregnancy were classified as complications and a continuation of the initial case. All episodes occurred during pregnancy and patients were considered as having HTG‐IPP in accordance with the revised Atlanta Criteria for acute pancreatitis [20], with corresponding severe HTG, and the absence of a more likely alternate cause for pancreatitis. Bedside index for severity in acute pancreatitis (BISAP) score was calculated as a measure of pancreatitis severity. No identified cases were excluded from the series after applying these criteria. Written informed consent was obtained from each of the patients for the purposes of this case study.

A literature review of previous cases of HTG‐IPP with an identified pathogenic variant was performed using OVID MedLine for English language publications with key terms ‘hypertriglyceridaemia’, ‘chylomicronaemia’, ‘pancreatitis’, and ‘pregnancy’ as well as pathogenic genes LPL, GPIHBP1, apolipoprotein A5 (APOA‐5), LMF1 and APOE.

## 3. Cases

### 3.1. Case One

34‐year‐old female of Taiwanese ethnicity, gravida 2 para 0 (G2P0), presented with severe epigastric pain and nausea at 25^+5^ weeks gestation. Pathology revealed an elevated lipase of 181 units/L, TG level of 87.3 mmol/L and total cholesterol of 25.6 mmol/L, indicating a diagnosis of acute pancreatitis secondary to severe HTG‐IPP. She was initially managed as nil by mouth and with an intravenous insulin and glucose infusion, before being transitioned to very low‐fat diet (VLFD), supplemental omega‐3 fatty acid (O3FA) and gemfibrozil, which she remained on throughout pregnancy. The patient was monitored with weekly TG testing with results ranging between 7.2 and 13.2 mmol/L. The pregnancy was complicated by insulin requiring gestational diabetes mellitus (GDM). At 37 weeks gestation following premature rupture of membranes, a healthy female baby weighing 3235 g was delivered by vaginal delivery. The baby had an Apgar score of 9/9, and was admitted to the neonatal intensive care unit (NICU) due to being febrile on Day 0 post‐partum. After 24 h of intravenous antibiotics, they were discharged home. Genetic testing performed post‐partum identified heterozygote mutations in both LPL (exon 6, variant c.835C >G, p.Leu279Val) and APOA‐5 (c.644T >C, rs662799), which are indicative of multifactorial polygenic chylomicronaemia. It was recommended on discharge for the patient to introduce medium‐chain TG (MCT) oil into her meals. Both the patient and her child were healthy and well on outpatient review 18 months post‐partum with her TGs in target on her regimen.

### 3.2. Case Two

32‐year‐old female of Vietnamese ethnicity, G1P0, with a previous history of GDM requiring insulin and three admissions with HTG‐induced pancreatitis in a non‐pregnant state presented for preconception counselling. Atorvastatin 20 mg daily and fenofibrate 145 mg daily were ceased preconception whilst low fat diet and O3FA 3 g three times a day (TDS) were continued. She presented at 19^+5^ weeks gestation with severe epigastric pain and nausea. Biochemistry revealed serum lipase 161 units/L, TG 71.8 mmol/L and TC 14.7 mmol/L. Resolution of symptoms and HTG was achieved over a period of 4 days with fasting and intravenous insulin and glucose infusion. Subsequently the patient was commenced on a low carbohydrate (LCHO) diet and VLFD, gemfibrozil 600 mg twice a day (BD), niacin 250 mg TDS and MCT oil 30 mL BD. Despite this, their TG remained above target (24.4–29.7 mmol/L), hence, simvastatin 10 mg daily was commenced after a multi‐disciplinary discussion with the patient.

The patient’s TG levels improved and she was discharged home for twice weekly TG‐testing but was readmitted 10 days later at 23^+6^ weeks gestation with asymptomatic HTG (37.8 mmol/L) despite good adherence with dietary measures. GDM was also diagnosed at 23 weeks gestation with a fasting blood glucose of 6.6 mmol/L. Continuous intravenous insulin and glucose infusion was recommenced, with simvastatin increased to 20 mg daily, niacin increased to 1 g TDS and gemfibrozil changed over to fenofibrate 145 mg daily. A double lumen tunnelled catheter was also inserted for therapeutic plasma exchange (TPE) and the patient referred for outpatient consideration of this at an alternate site where TPE was available.

Prior to receiving TPE the patient then represented at 26^+4^ weeks gestation with sacroiliac joint septic arthritis, tricuspid valve endocarditis and sepsis secondary to methicillin‐sensitive *Staphylococcus aureus* (MSSA) bacteraemia with the unused tunnelled catheter identified as the source of infection. This was removed, with the patient commenced on flucloxacillin therapy for 6 weeks as directed by the infectious diseases team. During their inpatient stay, HTG‐IPP reoccurred at 27^+4^ weeks (TG 14.3 mmol/L) and the patient ultimately required a daily intravenous insulin‐glucose infusion to maintain TG stability for the remainder of her pregnancy and was unable to be discharged. The patient received multiple 48–72 h continuous intravenous insulin‐glucose infusions before switching to an intermittent 11 h a day intravenous insulin‐glucose infusion for the remainder of pregnancy. Her simvastatin was switched to pravastatin 20 mg daily at 30^+3^ weeks gestation, with other agents unchanged. An underweight but otherwise healthy male of 1842 g was delivered by elective caesarean section in the setting of intrauterine growth restriction at 35^+5^ weeks gestation with an Apgar score of 9/9. The baby was initially in the NICU for management of low birth weight and hypothermia before discharging to the ward and then home at 21 days and both mother and baby were healthy and thriving at subsequent outpatient clinic follow up to 15 months. The mother remained on a low fat diet with daily fish oil, MCT oil and atorvastatin with fluctuating TG levels (1.6–13.1 mmol/L). Genetic testing identified a homozygous mutation in GPIHBP1 (exon 3, variant c.209C >G, p.Ser70Cys).

### 3.3. Case Three

36‐year‐old female of Vietnamese ethnicity, G2P1, with GDM managed with insulin presented to hospital at 35^+4^ weeks gestation with severe epigastric pain and a corresponding serum lipase of 426 units/L. Further aetiological testing was not undertaken initially. The patient was administered betamethasone for fetal lung maturation before undergoing emergency caesarean on Day 3 due to non‐reassuring cardiotocography, delivering a live female of 2380 g with an Apgar score of 9/9. The baby required a short NICU admission for management of hypoglycaemia. Bloods taken intra‐operatively revealed TG 118.1 mmol/L and TC 40.8 mmol/L. The patient was initially allowed to eat post‐operatively. Repeat lipid profiling at 24 and 48 h were improved but remained grossly deranged with ongoing abdominal pain leading to referral to endocrinology and subsequent fasting and commencement of a continuous insulin‐glucose infusion and subcutaneous heparin 5000 units BD for 7 days. She was then commenced on LCHO, VLFD, O3FA 2 g daily and niacin 500 mg daily. Serum TG was 9.4 mmol/L at Day 10 post‐partum and patient was discharged on an unchanged oral regime. Both patient and baby were thriving at follow‐up outpatient endocrinology reviews to 12 months with TG to target on O3FA, niacin and gradual return to unrestricted diet with TG to target. Post‐partum genetic testing identified a heterozygous mutation in APOA5 (c.644T >C).

### 3.4. Case Four

29‐year‐old female of Indian ethnicity, G2P1, with history of GDM as well as previous episodes of pancreatitis during an earlier pregnancy and in non‐pregnant state presented again with pancreatitis at 34 and 36 weeks gestation. The 2 episodes of HTG‐IPP were managed with fasting, intravenous insulin‐dextrose infusions, O3FA supplementation and subsequent VLFD with MCT oil supplementation. The patient proceeded with an elective caesarean section at 36^+3^ weeks gestation in the context of rising TG level at 51.6 mmol/L. There was no evidence of foetal distress. The infant was born healthy at a weight of 3420 g.

Following this pregnancy she had genetic testing with finding of polygenic chylomicronaemia due to a compound heterozygote with 2 GPIHBP1 variants, specifically c.295G >T, p.Glu99 ^∗^ and c.323C >T, p.Thr108Met. These were classified as pathogenic and likely pathogenic respectively at the time of reporting. There was therefore no scope for pre‐pregnancy counselling and optimisation and despite the diagnosis, she would go on to have further episodes of pancreatitis in subsequent pregnancies as well.

## 4. Discussion

### 4.1. Risk Factors and Co‐Morbid Associations

All four of our patients were found to have genetic mutations associated with HTG, and a summary of the four cases is provided in Table 1. These mutations were within GPIHBP1, LPL and APOA‐5, which are all loss of function mutations. FCS is rare with mutations in lipoprotein lipase implicated in over 90% of cases [21]. Homozygous LPL mutation is present in approximately one in one million patients, whereas heterozygous LPL mutation is present in approximately one in 500 patients [22] which may confer different risks for severity of HTG.

**Table 1:** Summary of pertinent details of patient cases described in the body of this case series.

Case number	Age (years)	Ethnicity	Genetic mutation	Peak triglyceride level (mmol/L)	Gestation at presentation with HTG‐IPP (weeks)
One	31	Chinese	Heterozygous for LPL. Exon 6. Variant c.835C >G Heterozygous for APOA5. c‐644T >C (rs662799)	87.3	25^+5^
Two	30	Vietnamese	Homozygous for GPIHBP1. Exon 3. Variant c.209C >G	71.8	19^+5^
Three	36	Vietnamese	Heterozygous for APOA5. Variant c.‐644T >C	118.1	35^+4^
Four	29	Indian	Compound heterozygous for GPIHBP1 variants c.295G >T, p.Glu99 ^∗^ c.323C >T, p.Thr108Met	51.6	34

In our cohort, both case one and three had the same genetic mutation of APOA5 with case one also involving a heterozygous mutation of LPL. Both cases involved only single admissions with HTG‐IPP and the shortest time in hospital which may suggest that the identified heterozygous mutations in c.644T >C (APOA5) and c.835C >G (LPL) are less pathological than others. Case four had recurrent presentations with acute pancreatitis in the presence of heterozygosity for two separate GPIHBP1 variants however management with medical therapy was relatively uncomplicated. Case two was the only case to have a homozygous mutation and this case was the most resistant to medical therapy and presented earlier in pregnancy, suggesting that the identified homozygous mutation to c.209C >G (GPIHBP1) in this case may confer a more severe genetic susceptibility to HTG and constitute an underlying diagnosis of familial chylomicronaemia in this patient with a more severe phenotype.

All of our cases were of Asian or South Asian descent, with two Vietnamese, one Chinese and one Indian patient. This suggests that those of Asian ethnicity may be more likely to have genetic mutations that increase the likelihood of HTG‐IPP and is in keeping with previous studies from China that found HTG to account for the majority of causes of acute pancreatitis in pregnancy [8, 23], whereas other studies in Europe and North America have found biliary obstruction and alcohol to be at least equally as common [7, 24]. In addition to genetics, this may be related to cultural differences in diet, alcohol intake or a combination of all these factors.

All of our patients developed co‐morbid GDM. Suboptimally controlled diabetes mellitus is a known secondary cause of HTG and is the most common secondary risk factor for HTG. Hyperglycaemia is thought to contribute to HTG by reducing the activity of LPL [25], providing evidence for insulin resistance as a key factor in the pathophysiology of their association [22]. Patients with higher levels of TG early in pregnancy were noted to have both a higher incidence and risk of developing GDM [26]. Similarly, waist circumference greater than 85 cm combined with an elevated TG level above 1.7 mmol/L was found to be associated with an increased risk of a positive oral glucose tolerance test and a diagnosis of GDM [27]. Lifestyle measures to address insulin resistance such as moderate to vigorous physical activity were associated with improved insulin sensitivity and decreased TG levels in overweight and obese pregnant women in the third trimester [28]. The association seen between these two conditions reinforces existing local guidelines for universal GDM screening in pregnant patients, noting a potentially higher risk in those with concurrent HTG.

### 4.2. Management of HTG‐IPP

There are no current clinical guidelines for the management of HTG‐IPP, and there have been no randomised trials conducted. There is a scarcity of evidence in this area, but available literature supports the use of fasting and subsequent introduction of VLFD, LCHO diet, nutritional supplementation (O3FA and MCT), parenteral nutrition, insulin, heparin and TPE [1, 22] for the management of HTG‐IPP. Dietary management is a key pillar of HTG management and a LCHO diet is less preferred in comparison to VLFD as it has been suggested to be counterproductive and induce lipolysis, leading to increased levels of glucose, free fatty acids and ketone levels. However, without strong evidence to support this hypothesis, general practical advice has revolved around reducing simple carbohydrate intake, increasing complex low glycaemic carbohydrates and reducing fat intake below 10% of the daily total caloric intake [22]. Niacin and fibrates are pharmacological options that have been used in previous case studies without teratogenic effects, with modest efficacy in managing but ultimately not preventing HTG‐IPP, perhaps relating to the severity of the individual cases described [1]. Statin medication has recently been re‐assessed as a viable ongoing treatment for HTG with mixed hyperlipidaemia pre‐conception and during pregnancy, due to historical concerns regarding congenital malformation risk, which later observational studies have not replicated [29]. It should be noted that congenital malformation risk was determined on animal studies and at higher doses than prescribed to humans for its lipid‐lowering effect [30]. Meta‐analyses indicating an association between statins and low birth weight, pre‐term birth [29] and spontaneous abortion [31] need to be considered against risk to the health of the mother secondary to severe HTG.

### 4.3. Genetic Testing

There are also no clinical guidelines to assist with determining the role of genetic testing, and potentially screening, in at risk populations for severe HTG, exacerbated in pregnancy as seen in our case studies. Rationalising genetic screening is particularly difficult in the setting of variants of unknown significance that may alter decision‐making despite a lack of evidence to support the pathogenesis of some mutations, with isolated case studies described for most. HTG can occur as a result of a collection of multiple small‐effect DNA variations, known as single nucleotide polymorphisms (SNPs), which in isolation may only slightly affect lipid levels, but when present in sufficient numbers can result in a phenotype that presents similarly to that of a single, large‐effect mutation. A summary of previous case studies and pathological variants or mutations resulting in familial or polygenic chylomicronaemia and hypertriglycaeridaemia with associated HTG‐IPP is detailed in Table 2. The identification of these SNPs through targeted next generation sequencing panels can be used to form polygenic risk scores (PRSs) to quantify the expected effect of these SNPs on a patient’s risk of HTG compared to a normotriglyceridaemic population [44]. A retrospective case–control study in academic lipid clinics used the assessment of rare, heterozygous pathogenic variants (PVs) and PRSs through the identification of multiple common, small‐effect SNPs. It was able to demonstrate an association between the presence of both PV and high PRS and a significantly increased risk of very severe HTG and acute pancreatitis [45]. With advances in genome‐wide association studies, a wider spectrum of SNPs associated with TG levels have been identified, and although largely non‐coding in nature with unclear clinical utility, a high prevalence of SNPs in severe HTG has been repeatedly demonstrated with strong statistical significance [46]. Targeted next generation sequencing of LPL and four associated co‐factor genes (LMF1, GPIHBP1, ApoC2, and ApoA5) is the preferred method of confirming a diagnosis of familial or polygenic chylomicronaemia [47, 48]. The current prohibitive cost considered against low disease prevalence in an Australian population renders screening economically unfeasible [48]. With further case identification, particular variants of pathogenic interest can be identified and screened for antenatally in patients with a moderate fasting HTG of more than 200 mg/dL (11.1 mmol/L) [45] in the absence of other causative factors such as uncontrolled hypothyroidism or alcohol misuse, to encourage proactive management and monitoring of HTG risk, especially in the pre‐conception period and during pregnancy where physiological exacerbation is expected. Confirmation of moderate HTG in this context would also be an appropriate opportunity to consider genetic testing in familial members for disorders of lipid metabolism. Guidelines surrounding the assessment of familial hypercholesterolaemia are robust, particularly with the introduction of novel agents such as proprotein convertase subtilisin/kexin type 9 (PCSK9) inhibitors, however this remains not to be the case for FCS and HTG [49].

**Table 2:** Summary of previous case studies and pathological variants/mutations resulting in familial or polygenic chylomicronaemia and hypertriglycaeridaemia with associated HTG‐IPP.

Variant	Genetic mutations
LPL	1. Compound heterozygote for two missense mutations p.I194T and p.R243H [1] 2. Compound heterozygote for Leu252Arg and Ala261Thr mutations [9] 3. Heterozygote for Asn291Ser substitution [9] 4. Heterozygote for Trp382Stop mutation [9] 5. Homozygote for Ser172Cys mutation [9] 6. Compound heterozygote for p.A98T and p.L279V [14] 7. Compound heterozygote for Gly242Lys and Leu252Val [15] 8. Combination of two‐point nonsense mutations—^221^ *∆G*⟶Stop, ^447^Ser⟶Stop [32] 9. Homozygote for nonsense mutation in exon 8 (TGG^1401^ ⟶ TGA/Trp^382^ ⟶ Stop) [33] 10. Heterozygote for missense mutation p.His210Leu (c.629A >T) [34] 11. Heterozygote for Glu421Lys [23] 12. Homozygote for Trp86Arg [35] 13. Two sisters, compound homozygote for p.G236Gfs ^∗^15 deletion and p.G215E missense mutation [36] 14. Two sisters, heterozygote Gly188Glu and ApoE e3/e3 [12] 15. Homozygote for c.1019‐2A >T [37]
GPIHBP1	1. Compound heterozygote for p.A98T (in LPL) and p.C14F (in GPIHBP1) [14] 2. Homozygous frameshift mutation c.48_49insCGGG (p.P17A fs ^∗^22) [38]
APOA‐5	1. Homozygote for p.G185C [14]
LMF1	1. Homozygous nonsense variant c.697C >T, p.Arg233Ter [39]
APOE	1. e2/e3 genotype with reduction in ApoE protein [40] 2. Combined heterozygote for pSer19Trp (APOA‐5) and pCys130Arg (APOE allele E4) [41] 3. Combined heterozygote e2/e3 ApoE genotype and Glu116Asp (LPL) [42] 4. Compound heterozygote for e4/e2 genotype of ApoE [43]

### 4.4. Risk Optimisation in the Pre‐Conception Period

In patients with pre‐existing genetic HTG or FCS, as well as those that are newly diagnosed such as with our cases, optimisation of TG levels prior to pregnancy is important to reduce the risk of HTG‐IPP. Within current clinical practice, pharmacotherapy such as statin therapy is generally ceased prior to or at the time of conception. This places a larger emphasis on the role of lifestyle modification, education and adherence to a VLFD in managing worsening HTG during this period of patient care. There are no clear recommendations for screening of HTG itself in pregnancy, despite its association with poor pregnancy outcomes. Existing recommendations discuss screening general lipid profiles prior to pregnancy if considered an at‐risk group, such as with a familial or personal history dyslipidaemia, obesity with other risk factors, a history of pancreatitis or physical signs suggestive of HTG, however, do not detail further investigation into potential underlying genetic causes [22]. Based on this case study series, given the lack of evidence otherwise, an anecdotal approach in a compound heterozygous LPL patient of monitoring TG levels every two to 3 weeks in the outpatient setting with short‐term hospital admissions for management of elevated levels or clinical exacerbations has been described [1]. The strength of association between homozygosity and an increased severity of HTG and peak TG levels in pregnancy has not been clearly reported, including through our case series, and therefore an increased frequency of monitoring in homozygous patients is not indicated based on current evidence. As detailed in Table 3, chronic and ongoing therapy for HTG in pregnancy is relatively safe, but still recommended with caution. Niacin at 18 mg daily is recommended in pregnancy but safety at lipid‐lowering doses in the range of 1500–3000 mg daily is unclear [54]. Fibrates are known to cross the human blood‐brain barrier [54], however, have not shown teratogenicity in available case reports [1]. Gemfibrozil has only been studied in small populations and as such, confident conclusions regarding their safety are unable to be made. Statins are commonly used outside of pregnancy for general management of HTG and as mentioned, recent meta‐analyses have provided some reassurance of their safety in the pre‐conception period, but their use in pregnancy remains controversial. Statins do not appear to have a role in acute HTG‐IPP as they do not have a significant TG lowering effect however may be considered in rarer instances, where LDL reduction is also desirable. Pharmacological therapy aside, education surrounding the management of lifestyle factors such as exercise, glycaemic control and dietary counselling early during the pre‐conception process is recommended in all patients at risk [54].

**Table 3:** Summary of current management options for acute and chronic hypertriglyceridaemia, in the context of pregnancy.

Treatment	Pathophysiology
Acute ‐ intravenous insulin and glucose	Insulin is a rapid and potent LPL activator which causes immediate and significant reductions in serum TG [50] Glucose is given concurrently to prevent hypoglycaemia, without a direct effect on triglyceridaemia
Acute ‐ heparin	Transient effect of activating LPL which reduces circulating TGs There is also evidence that following an initial rise in LPL activity, there is a subsequent depletion in LPL with sustained heparin use which can result in rebound HTG [50]
Acute ‐ therapeutic plasma exchange (TPE)	Physical removal of lipoproteins from the blood [51]
Chronic/ongoing ‐ low fat diet (LFD)/very low fat diet (VLFD)	Reduced exogenous substrate lowers TG levels
Chronic/ongoing ‐ omega‐3 fatty acids (O3FA)	Inhibits hepatic lipogenesis, stimulates fatty acid oxidation in the liver and skeletal muscle, and may also stimulate LPL [52]
Chronic/ongoing ‐ medium chain triglycerides (MCT) oil	Absorbed directly from bloodstream by the liver for rapid oxidation, by‐passing chylomicrons and adipose tissue, reducing the need for LPL [53] Oral supplementation and replacement of triglycerides to prevent essential fatty acid deficiency in setting of VLFD and support foetal neurocognitive development [54]
Chronic/ongoing ‐ fibrates	Peroxisome proliferator‐activator receptor alpha (PPAR‐alpha) working through various mechanisms; metabolism of TG‐rich lipoproteins and upregulation of LPL activity [55]
Chronic/ongoing ‐ niacin	Directly and noncompetitively inhibits hepatocyte diacylglycerol acyltransferase–2, a key enzyme for TG synthesis Inhibition of TG synthesis resulting in accelerated intracellular hepatic apo B degradation and the decreased secretion of VLDL and LDL particles [56]
Chronic/ongoing ‐ statins	Lowers LDL levels by inhibiting HMG‐CoA reductase activity leading to decreases in hepatic cholesterol content resulting in an up‐regulation of hepatic LDL receptors, which increases the clearance of LDL [57]

## 5. Conclusion

In conclusion, HTG‐IPP is a rare but potentially serious condition. There is a paucity of high‐quality evidence to guide management and clinical treatment guidelines remain lacking. Dietary changes, O3FA, MCT, insulin and TPE are all safe treatment options in this cohort with existing safety data for the use of fibrates and niacin. In those at known high or increased risk of HTG‐IPP or previous refractory HTG, early referral to a tertiary centre which has the capacity for TPE should be considered; however, we have demonstrated that intravenous insulin, VLFD with supplementation with MCT oil, fibrates and niacin can effectively manage most cases of HTG‐IPP.

Our case series is the largest publication of HTG‐IPP with genomics included. It has several strengths, such as the inclusion of a literature review to guide recommendations for management of HTG and HTG‐IPP, detailed genetic mutation data to categorise mutations and identify risk factors for HTG and discussion surrounding the current evidence base in support of our experience with our patients. A weakness of our case series is the relatively small number of patients, with four case studies; however, the characteristics of these were able to supplement information provided by previous cases found in the literature. We recognise that this, combined with the level of evidence provided by a case series, restricts us from making major conclusions regarding genetic hypertriglycaemia and acute pancreatitis in pregnancy. Further case studies and genomics research are needed to add to currently available evidence and increase our understanding and capacity in predicting risk of severe gestational HTG. Pre‐conception optimisation of TGs with appropriate diet and pharmacotherapy management plans that are tailored to the individual and regular monitoring in pregnancy can then reduce the incidence and disease burden associated with HTG‐IPP and HTG.

## Author Contributions

Dev Kevat and I‐Lynn Lee conceived the concept of the manuscript and supervised the project. Emily Gianatti, I‐Lynn Lee and Dev Kevat contributed case reports from clinical experience and provided feedback to shape the final manuscript. Nicholas Adams and Christopher Son Nguyen gained patient consent for case report write‐up and performed a preliminary literature review. Christopher Son Nguyen performed a detailed literature review and wrote the final manuscript with input from all authors.

## Funding

No funding was received for this manuscript.

## Disclosure

All authors have read and approved the final version of the manuscript. Christopher Son Nguyen had full access to all of the data in this study and takes complete responsibility for the integrity of the data and the accuracy of the data analysis.

## Consent

Written consent from patient was obtained for these case studies.

## Conflicts of Interest

The authors declare no conflicts of interest.

## Data Availability

Data sharing is not applicable to this article as no new data were created or analysed in this study.
